# Biopsy Induced Arteriovenous Fistula and Venous Stenosis in a Renal Transplant

**DOI:** 10.1155/2015/313610

**Published:** 2015-08-25

**Authors:** Sridhar R. Allam, Balamurugan Sankarapandian, Imran A. Memon, Patrick C. Nef, Tom S. Livingston, George Rofaiel

**Affiliations:** ^1^Tarrant Nephrology Associates, 1001 Pennsylvania Avenue, Fort Worth, TX 76104, USA; ^2^Division of Transplant Nephrology, Fort Worth Transplant Institute, Plaza Medical Center, 900 Eighth Avenue, Fort Worth, TX 76104, USA; ^3^Department of Interventional Radiology, Plaza Medical Center, 900 Eighth Avenue, Fort Worth, TX 76104, USA; ^4^Division of Transplant Surgery, Fort Worth Transplant Institute, Plaza Medical Center, 900 Eighth Avenue, Fort Worth, TX 76104, USA

## Abstract

Renal transplant vein stenosis is a rare cause of allograft dysfunction. Percutaneous stenting appears to be safe and effective treatment for this condition. A 56-year-old Caucasian female with end stage renal disease received a deceased donor renal transplant. After transplant, her serum creatinine improved to a nadir of 1.2 mg/dL. During the third posttransplant month, her serum creatinine increased to 2.2 mg/dL. Renal transplant biopsy showed BK nephropathy. Mycophenolate was discontinued. Over the next 2 months, her serum creatinine crept up to 6.2 mg/dL. BK viremia improved from 36464 copies/mL to 15398 copies/mL. A renal transplant ultrasound showed lower pole arteriovenous fistula and abnormal waveforms in the renal vein. Carbon dioxide (CO_2_) angiography demonstrated severe stenosis of the transplant renal vein. Successful coil occlusion of fistula was performed along with angioplasty and deployment of stent in the renal transplant vein. Serum creatinine improved to 1.5 mg/dL after.

## 1. Introduction

Renal transplant vein stenosis is a rare cause of allograft dysfunction. It can result from damage to the vein during organ procurement or from surgical complications like hematoma, lymphocele, or torsion of renal vein. Other reported etiologies include allograft rejection [1], renal vein thrombophlebitis from adjacent infected fluid collection [2], high-pressure turbulent flow in the presence of arteriovenous fistula in the renal allograft [3], external compression from the crossing iliac artery [4], preexisting renal vein stenosis in the donor kidney [5], or idiopathic [6]. Renal transplant vein stenosis should be considered in the differential diagnosis of unexplained allograft dysfunction. The use of balloon venoplasty and/or stent placement seems to be a safe and effective approach. Herein, we report a case of biopsy induced arteriovenous fistula that led to renal transplant vein stenosis and allograft dysfunction.

## 2. Case Presentation

A 56-year-old Caucasian female with end stage renal disease due to hypertension, who was on hemodialysis for about 5 years, received 1A 2B 1DR antigen mismatch deceased donor renal transplant. The donor was a 50-year-old lady that died of streptococcal meningitis with terminal creatinine of 0.8 and kidney donor profile index score of 47%. The cold ischemia time was 3 hours and 22 minutes and warm ischemia time was 29 minutes. Surgery was uncomplicated with implantation of kidney in the right lower quadrant of abdomen with end-to-side anastomosis to external iliac vessels. Patient received thymoglobulin (6 mg/kg total) induction therapy followed by triple immunosuppressive regimen of tacrolimus, mycophenolate, and prednisone. Patient had immediate graft function with improvement in serum creatinine to a nadir of 1.2 mg/dL. During 3-month posttransplant office visit, her serum creatinine increased to 1.6 mg/dL. She was noted to have BK viremia of 13920 copies/mL. Mycophenolate was reduced from 720 mg twice daily to 360 mg twice daily. About 2 weeks later, serum creatinine further increased to 2.2 mg/dL. Blood BK PCR increased to 36464 copies/mL. Renal transplant ultrasound showed elevated resistive indices. Renal transplant biopsy was performed that showed focal severe acute tubular injury with isolated small foci of inflammation surrounding the affected tubules. There was no evidence of tubulitis, arteritis, or significant interstitial inflammation. There was mild tubular atrophy and interstitial fibrosis. SV40 stain showed clusters of weak to very strong staining of tubular epithelial nuclei. Mycophenolate was discontinued and tacrolimus dose was adjusted to maintain trough level of 3 to 5.

**Figure 1:**
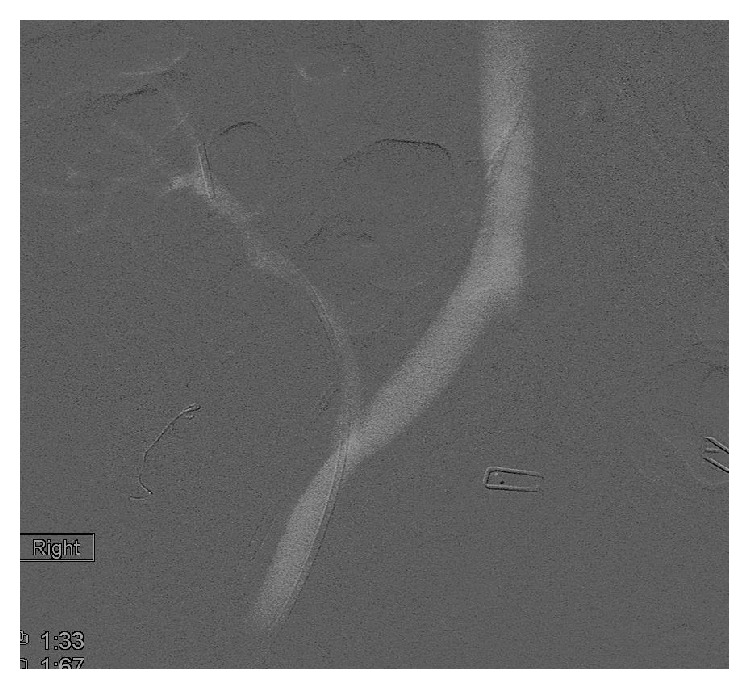
CO_2_ angiogram showed high-grade stenosis of renal transplant vein.

**Figure 2:**
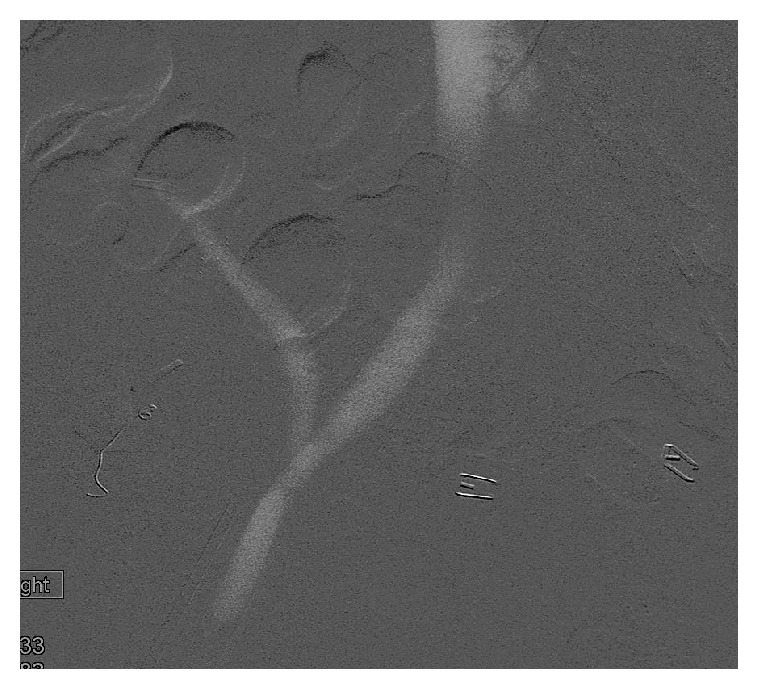
CO_2_ angiogram obtained after placement of stent in renal transplant vein showed no residual stenosis.

Over the next month, her serum creatinine increased to 3.1 to 3.5 mg/dL range. During her fifth posttransplant month office visit, she reported progressive oliguria and lower extremity edema. She was noted to be fluid overloaded on exam. Serum creatinine further increased to 6.2 mg/dL. Random urine protein by creatinine ratio showed nephrotic range proteinuria with a ratio of 4.5, which was a significant increase from 1.1 a month prior. She was hospitalized at that point and was dialyzed on two consecutive days for fluid overload. Blood BK PCR actually improved to 15398 copies/mL. A renal transplant ultrasound showed lower pole arteriovenous fistula, increased velocities, and abnormal waveforms in the renal vein. Given dialysis dependent allograft dysfunction despite improvement in BK viremia and abnormal findings of the ultrasound, we proceeded with interventional radiological procedures. Common iliac angiography with CO_2_ demonstrated patent common and external iliac arteries. There was a widely patent end-to-side renal artery anastomosis. Arteriovenous fistula was demonstrated in the lower pole of the transplant kidney with early filling of the renal vein. There was severe stenosis of the main renal vein. Successful coil occlusion of fistula was performed using a 3 mm × 50 mm microcoil. Then, renal venography using CO_2_ was performed that confirmed a high-grade stenosis of the renal vein for a length of approximately 30 mm (Figure 1). Angioplasty was initially performed with 8 mm × 4 cm balloon followed by 10 mm × 4 cm balloon. Repeat venography again demonstrated moderately severe stenosis of the renal vein. Then, 8 mm × 30 mm self-expanding stent was deployed across the renal vein stenosis. Repeat venography demonstrated no significant residual stenosis (Figure 2). Percutaneous renal transplant biopsy performed at the same time showed moderate to severe acute tubular injury with mild interstitial infiltrate consisting of lymphocytes and plasma cells. There was mild arteriosclerosis, interstitial fibrosis, and tubular atrophy (about 10%). There was positive tubular epithelial nuclear staining for SV40. In addition, there was extensive interstitial edema, likely a consequence of significant venous outflow obstruction. Her urine output significantly improved after renal vein stenting and dialysis was discontinued after a total of three dialysis sessions. Serum creatinine eventually stabilized around 1.5 mg/dL range. Patient was given oral anticoagulation combined with antiplatelet therapy Aspirin 81 mg daily for 6 months after the procedure followed by long-term administration of Aspirin 325 mg daily.

## 3. Discussion

This patient's course is consistent with biopsy induced arteriovenous fistula that likely led to renal vein stenosis in the allograft. Although not reported in the literature before, another potential reason for development of venous stenosis in our patient could be BK virus induced inflammation. Progressive rise in serum creatinine despite improvement in BK viremia/nephropathy, extensive interstitial edema on biopsy that is indicative of venous outflow obstruction, and prompt improvement in allograft function following renal vein stenting support venous stenosis as the predominant etiology of her allograft dysfunction. A series of 8 cases of renal transplant vein stenosis causing allograft dysfunction was first reported in 1991 [3]. Of these 8 cases, 5 were causally related to development of arteriovenous fistula following renal transplant biopsies. Arteriovenous fistula causes high-pressure turbulent blood flow in the vein that may lead to spasm and stenosis of vein over time. Progression of venous stenosis can lead to complete occlusion and allograft loss [3]. There are no specific clinical symptoms or signs to suspect allograft dysfunction from renal vein stenosis. It can present weeks to years after renal transplantation. Diagnosis can be made by duplex ultrasound in some cases by visualization of reduced caliber and increased velocities in the vein. CT and MR angiography are other imaging modalities that can be used for diagnosis [2, 5]. Conventional or CO_2_ based venography may be required to diagnose or confirm renal vein stenosis suggested by other imaging modalities. Once the diagnosis is confirmed by venography, the balloon venoplasty and stent placement can be performed at the same time. This percutaneous technique holds promise by successful treatment of this condition and avoidance of open surgical procedure.

In summary, renal transplant vein stenosis should be considered in the differential diagnosis of unexplained allograft dysfunction. Percutaneous venoplasty and/or stenting appear to be safe and effective treatment for this rare condition.
